# The Pathogenetic Role of the HGF/c-Met System in Papillary Carcinoma of the Thyroid

**DOI:** 10.3390/biomedicines2040263

**Published:** 2014-10-24

**Authors:** Luigi Ruco, Stefania Scarpino

**Affiliations:** Department of Clinical and Molecular Medicine, Pathology Unit, Sant’Andrea Hospital, Sapienza University, 00189 Rome, Italy; E-Mail: luigi.ruco@uniroma1.it

**Keywords:** HGF/Met, Papillary carcinoma, thyroid

## Abstract

The *MET* oncogene encodes for Met protein, a trans-membrane tyrosine kinase identified as the high affinity receptor for hepatocyte growth factor (HGF). Immunohistochemical studies have demonstrated that Met protein is intensely expressed in tumor cells of >95% cases of thyroid papillary carcinoma. High density of Met protein in tumor cells is the result of increased transcription of a normal *MET* gene, probably due to a combination of intracellular and extracellular signals. Over-expression of Met protein is more pronounced at the invading front of the tumor and can profoundly affect the tumorigenesis of papillary carcinoma of the thyroid. In fact, Met protein-positive papillary carcinoma cells are highly responsive to hepatocyte growth factor (HGF), which is effective in stimulating tumor cell adhesion, migration and invasiveness. In addition, HGF stimulation of papillary carcinoma of the thyroid (PTC) cells causes up-regulation of COX-2 and down-regulation of CD82/KAI-1; both these molecules have a major role in controlling tumor cell invasiveness. Finally, HGF stimulation of tumor cells may significantly affect the tumor microenvironment. In fact, HGF induces tumor cells to release chemokines active in the recruitment of dendritic cells, and is involved in regulating the production of proangiogenic factors.

## 1. Introduction

Papillary carcinoma of the thyroid (PTC) is the most common malignant tumor of the thyroid. Mean age of patients is 30–50 years; females are more affected than males (3:1). In spite of its well-differentiated histology, the tumor is highly invasive. In fact, a multifocal involvement of the thyroid gland is present in 18%–22% of the patients. Moreover, about 35% of the patients have clinically evident tumor metastasis at the time of diagnosis; this percentage rises to 50% after histological examination of lymph nodes. Hematogenous metastases are relatively rare; they are present in 4%–14% of patients and the lung is the most common site. In spite of its high diffusiveness, PTC has an excellent prognosis; in fact, >95% of patients are alive and well after five years [1].

In the last 10 years, evidence has accumulated that over-expression of Met protein is a distinguishing feature of almost every case of well-differentiated papillary carcinoma. Increased expression of the protein is probably due to enhanced transcription of the *MET* gene and/or to post-transcriptional mechanisms [2]. Thus, the possibility exists that dysregulation of *MET* is the final result of different molecular pathways capable of inducing thyroid cell transformation.

Dysregulation of *MET* causes marked accumulation of Met protein in tumour cells. Over-expression of Met protein can profoundly affect the tumorigenesis of papillary carcinoma of the thyroid because crucially involved in the control of the “invasive growth”. In fact, Met protein-positive papillary carcinoma cells are highly responsive to hepatocyte growth factor (HGF) which is effective in stimulating tumor cell adhesion, migration, invasiveness and induces the synthesis and release of chemokines and growth factors [3,4].

## 2. Papillary Carcinoma of the Thyroid and *MET* Expression

The molecular mechanisms involved in malignant transformation of PTC have been extensively investigated [2]. Different types of RET rearrangements are present in 20%–50% of cases [5]; point mutations of BRAF (V599E) have been detected in 30%–40% of cases [6,7,8]. It is of interest that RET rearrangements and BRAF mutations are mutually exclusive, thus suggesting that they represent founder molecular events in malignant transformation.

In the early 1990s it was demonstrated that over-expression of *MET* gene and of Met protein are distinguishing features of almost every case of PTC [9]. Gene expression profile studies have demonstrated that MET gene is one of the 23 genes, which is significantly more expressed in PTC [10]; increased gene transcription resulted in marked over-expression of Met protein (up to 100-fold) [11]. This observation was confirmed in immunohistochemical studies on tissue sections [12,13,14,15,16] where it was found that 75%–100% of PTC, including all the histological variants, over-express Met protein with a typical basolateral expression on the cell membrane [17]. Met protein is not present in normal thyroid follicles, but may become detectable in some inflammatory conditions, such as chronic lymphocytic thyroiditis. These findings are consistent with the possibility that expression of Met protein is finely regulated in normal thyroid cells and is profoundly dysregulated in PTC cells. These observations raise the possibility that different transforming events may activate a final common pathway leading to dysregulation of *MET* transcription and, hence, to Met protein over-expression.

## 3. Molecular Mechanisms of *MET* Dysregulation in Papillary Carcinoma of the Thyroid (PTC)

**Figure 1:**
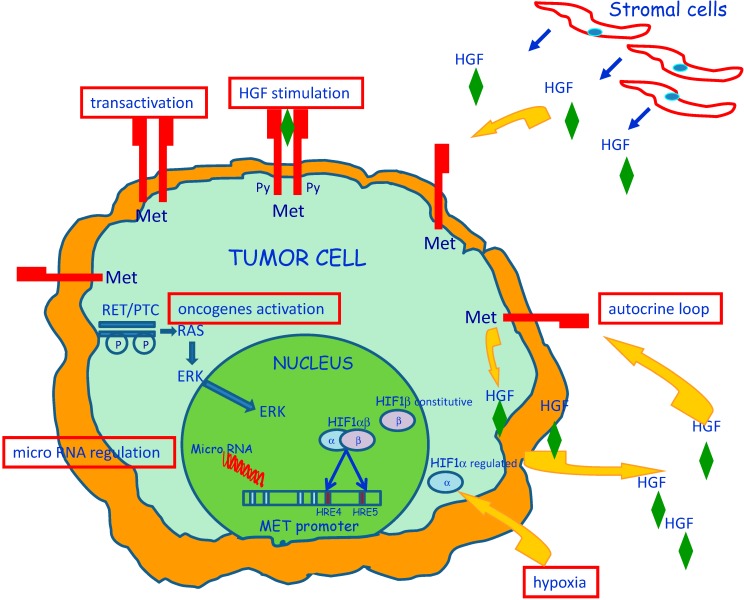
Transforming events that induce Met protein over-expression in papillary carcinoma of the thyroid. Increased *MET* transcription can be due by a combination of intracellular and extracellular factors as summarized in the image. Dysregulation or mutation of other oncogenes, may contribute to regulate Met protein levels and also *MET* promoter activity modification induced by methylation or interaction with microRNAs can have a crucial role. Up-regulation of *MET* transcription can be caused also by extracellular factors, such as HGF stimulation and hypoxia conditions.

The c-MET proto-oncogene encodes the Met protein, the high affinity receptor for HGF [18,19,20,21]. Met protein is a 190 kD heterodimer composed of two disulphide-linked chains: an extracellular 50 kD α-chain and a transmembrane 145 kD β-chain, with tyrosine kinase activity. In PTC, Met protein over-expression is not associated with amplification or rearrangements of the *c-MET* gene; moreover, the Met protein produced by tumor cells did not show major structural alterations [22]. The absence of any primary structural abnormality of the *MET* gene is consistent with the possibility that over-expression of the protein is dependent on enhanced transcription of a normal *MET* gene [23] (Figure 1).

Increased *MET* transcription can be due to dysregulation or mutation of other oncogenes, such as *RAS* [24], *ETS* [25], *RET* [26], and *BRAF* [27]. In fact, it has been shown that introduction of activated RAS and RET in normal thyroid cells results in over-expression of Met protein [24]; moreover, it was found that a consistent number of PTC cases have an activating mutation of BRAF that also cause signal transduction through the RET–RAS pathway.

Hypomethylation of the promoter may favor increased transcription of some oncogenes. As the *MET* promoter is a 697 bp 50-untranslated region that contains a typical CpG island spanning, with a frequency of CpGs 10-fold greater than in the total gene, the possibility was explored that an altered methylation status of the *MET* promoter is involved in the abnormal expression of Met protein in TPC [28]. MET transcription was investigated through the analysis of the methylation status of 43 CpGs in six cases of papillary carcinoma; no evidence of methylation was found in any of the analyzed CpG, suggesting that molecular mechanisms other than hypomethylation are responsible of high expression of *MET* gene in PTC.

MicroRNAs (miRNA) play a critical role in several biological processes and also in human cancers where they can act either as oncogenes, down-regulating tumor suppressor genes, or as onco-suppressors, targeting molecules critically involved in promotion of tumor growth. Different studies identified miRNAs (miR-34b, miR-34c, miR-1, miR-199a and miR-410), which negatively regulate the expression of *MET* [23,29]. Among these, the miR-199 family, including mature miR-199a-5p, miR-199a-3p, as well as miR-214, stands out as significantly down-regulated in PTC [29,30]. miR-199a-3p restoration in PTC cells reduces Met and mTOR protein levels, and impairs migration and proliferation of PTC tumor cells.

Increased MET transcription can be caused also by extracellular factors. Stimulation of PTC cells with HGF, the high affinity ligand for Met protein, may cause up-regulation of *MET* transcription [31]. In two different studies [3,15], HGF was detected in the tumor stroma and in the cytoplasm of some tumor cells, raising the possibility that an HGF/Met autocrine loop can be activated in PTC cells. Moreover, it was emphasized that HGF staining was present in a small fraction of tumor cells located at the invading front of the tumor [3] and was colocalized with PTC cells characterized by enhanced expression of Met protein. It seems possible that HGF/Met interactions in the leading front of the tumor may have a major role in favoring invasiveness.

It has been published that increased *MET* transcription can be induced by hypoxia [32]. It was speculated that increased cell motility may facilitate tumor cells to reach capillary vessels, which represent a source of oxygen. Hypoxia causes increased transcription of hypoxia inducible factor-1 (HIF-1). It was reported that HIF-1 has two binding sites on the MET promoter and is capable of increasing transcription of the gene in several distinct types of cancer cell line [32]. PTC is often characterized by the presence of fibrosis, necrosis and/or cystic degeneration. In a study of 64 cases of PTC, the levels of RNA transcripts for HIF-1 were (4.5 ± 3)-fold higher in the tumor tissue than in the surrounding normal thyroid tissues [33]. Microdissection of tumor cell nests from the tumor-invading front revealed that the levels of RNA transcripts for MET/HIF were higher than in the center of the tumor, thus, suggesting that HIF-1 may be one of the factors that contribute to the increased MET transcription in PTC.

## 4. Met Protein Over-Expression Facilitates Invasiveness of PTC

**Figure 2:**
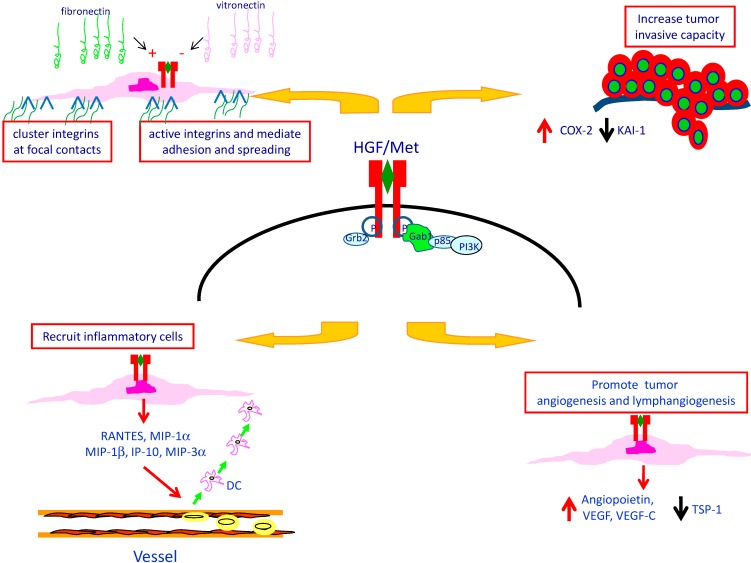
The Met/HGF invasive growth program. HGF activates the specific receptor Met inducing its dimerization. Once activated, Met recruits multiple adaptors and transducers on its multifunctional docking site. Specific pathways lead to different biological responses, which result in invasive growth. In papillary carcinoma of the thyroid, HGF stimulates the adhesion of cells to ECM components inducing firm attachment and spreading and enhancing migratory and invasion capacity of PTC cells. The Met/HGF interaction may interfere with tumor microenvironment, promoting tumor vascularization and releasing chemotactic factors involved in the recruitment of inflammatory cells into the tumor.

Activation of Met protein triggers a broad spectrum of biological responses through its multifunctional docking site (Figure 2). When phosphorylated, the two tyrosines in the receptor tail recruit numerous intracellular adaptors interacting with their SH2 domains [34]. The binding with the receptor can be either direct, such as for Shc [35], Src, Grb2, and the p85 regulatory subunit of PI3K [34], or indirect through the scaffolding protein Gab1 [36]. HGF/Met interaction controls a genetic program known as “invasive growth”, which involves as critical steps cell adhesion, migration, and trespassing of basement membranes [37,38].

It has been demonstrated that HGF stimulates the adhesion of PTC cells to several ECM components probably through multiple integrin activation [39]. In fact, stimulation of tumor cells with HGF causes clustering of integrins at focal contacts, mediates firm attachment and spreading, but does not affect their quantitative expression at membrane level [40]. The existence of a cooperative mechanism between adhesion molecules and Met protein is also suggested by the observation that adhesion of tumor cells to ECM components, by itself, induces Met protein activation, even in the absence of HGF [41].

HGF or agonistic monoclonal antibodies against Met protein stimulate the migratory capacity of PTC cells; in fact, they act as chemoattractants for tumor cells in an *in vitro* invasion assay and induce migration of papillary carcinoma cells through nucleopore filters coated with matrigel [42]. HGF stimulation causes prompt phosphorylation of Met protein and its action is more pronounced in PTC cells than in the corresponding normal thyroid cells obtained from the same patient.

The migratory capacity of PTC cells is profoundly influenced by the composition of ECM. In fact, in *in vitro* experiments it was shown that filters coated with FN were very effective in supporting the migratory response, whereas VN had an inhibitory effect [43]. When the distribution of ECM components was investigated in tissue sections of PTC it was found that VN is abundant in the basement membranes of normal follicles, whereas oncofetal FNs are present in the basement membranes and in the tumor stroma of papillary carcinoma [44,45]. Thus, HGF-stimulated papillary carcinoma cells might take advantage of a favorable ECM microenvironment, rich in oncofetal FN and poor in VN, to display their marked invasive behavior.

Cyclo-oxygenase 2 (COX-2) plays a major role in controlling invasiveness of tumor cells in many human neoplasms [46]. A marked increase in *COX-2* mRNA levels was observed in 8/8 primary cultures of TPC after HGF stimulation. Inhibition of COX-2 in TPC cells by the specific COX-2 inhibitor NS-398 significantly reduced the migration and invasiveness of tumor cells, but did not alter cell proliferation [47]. Analysis of microdissected samples of the tumor, and of the paired normal thyroid tissue, showed that mRNA transcripts for COX-2 were significantly higher in the tumor. Moreover, COX-2 was not homogeneously expressed throughout the tumor, but was significantly higher at the tumor invasion front.

COX-2 up-regulation was associated with down-regulation of KAI-1/CD82 [48], a metastasis suppressor molecule that has been associated with the metastatic potential of different solid tumors. Inhibition of COX-2 with NS398 is associated with an up-regulation of *KAI-1/CD82* RNA. The existence of a possible relation between COX-2 and KAI-1/CD82 was confirmed by the presence of an inverse correlation in the expression of the two genes in 55 tumor samples of PTC. Furthermore, in 36 of 55 cases, tumor areas contained lower levels of *KAI-1/CD82* RNA as compared with the corresponding normal tissue. PTC cases with low expression of *KAI-1/CD82* RNA by tumor cells were more often associated with extrathyroid extension of the disease and with lymph node metastasis. The relevance of KAI-1/CD82 in PTC cell invasiveness was confirmed in *in vitro* and *in vivo* experiments. In fact, re-expression of KAI-1/CD82 in PTC cells was associated with a significant reduction in their migratory and invasive capacity; nu/nu mice injected with KAI-1/CD82-transfected K1 TPC cell line developed fewer and smaller metastases, as compared with mice injected with vector-transfected K1 cells.

## 5. Met Protein Over-Expression May Influences the Tumor Microenvironment in PTC

Development of human cancers results from the cross-talk between cancer cells and the tumor microenvironment, which consists of ECM, blood vessels, inflammatory cells, fibroblasts and tumor-associated macrophages [49]. Evidence has been provided that HGF/Met protein interaction is one of the molecular mechanisms promoting tumor vascularization in PTC. In fact, it was shown that PTC cells contain RNAs of angiogenic factors, including angiopoietin, vascular endothelial growth factor (VEGF), and VEGF-C, and that HGF stimulation of PTC cells causes a five-fold increase in the amount of VEGF released in culture supernatants [4]. Moreover, HGF down-regulates the expression of Thrombospondin-1 (TSP-1), a potent inhibitor of angiogenesis. The poor production of TSP-1 in PTC was confirmed in tissue sections of the tumor. In fact, immunostaining for TSP-1 was generally faint, was associated with the tumor fibrous stroma, and was more intense in areas adjacent to the basal membrane of PTC cells. Moreover, *TSP-1* mRNA levels were significantly lower in tumor tissue as compared to the corresponding normal thyroid tissue. These findings are in line with the observation that HGF stimulation of primary cultures of PTC cells caused a marked decrease in TSP-1 at mRNA and at the protein level as well.

Papillary carcinoma of the thyroid is associated with a chronic inflammatory reaction in about 30% of cases [50,51]. Inflammatory cells are often aggregated to form an organized lymphoid tissue, defined tumor-associated lymphocytic thyroiditis, which is directed against the tumor and spares the peritumoral normal thyroid. Lymphocytic thyroiditis has a direct influence on tumor prognosis. In two independent studies, it has been demonstrated that the presence of lymphocytic thyroiditis is associated with a statistically significant improvement in overall survival and in cancer recurrence rates [1,52]. Moreover, a significantly greater proportion of patients with lymphocytic thyroiditis belonged to lower pathological tumor-node-metastasis stages, suggesting that the inflammatory reaction is effective in containing growth and spreading of tumor cells [52,53].

Macrophages and dendritic cells have a pivotal role in the organization of the tumor-associated inflammatory response [54]. Tissue distribution of dendritic cells was investigated in eight cases of PTC using immunohistochemistry. Most dendritic cells had an immature phenotype (CD1a^++^, CD11c^+^, CD40^+^, CD86^−^, HLA-DR^−^) and were located at the invasion edge of the tumor. This pattern of distribution was profoundly different from that of CD68^+^ macrophages, which were evenly distributed throughout the tumor. The ability of tumor cells to release chemotactic factors active on dendritic cells was investigated in primary cultures of the same cases. It was found that papillary carcinoma cells were active in releasing chemotactic activity, that HGF or interleukin (IL)-1β induced a four-fold increase in the amount of chemotactic activity released, and that normal thyroid cells obtained from the same patients were as effective as tumor cells. Characterization of chemokines at RNA level revealed that unstimulated cells contain large amounts of IL-8 and monocyte chemotactic protein (MCP)-1 RNAs, and that stimulation with HGF or IL-1β induced RNAs for regulated upon activation normal T expressed and secreted (RANTES), macrophage inflammatory protein (MIP)-3α, interferon-γ-inducible protein 10 (IP-10), and, to a lesser extent, MIP-1α and MIP-1β. Our observations are consistent with the possibility that HGF stimulation of Met + PTC cells is one of the molecular mechanisms involved in the recruitment of dendritic cells into the tumor.

## 6. Conclusions

*MET* gene is over-expressed in >95% PTC and is associated with over-expression of Met protein. *MET* gene over-expression is not associated with amplification or rearrangements of the gene; moreover, the Met protein produced by tumor cells has no major structural alterations. Thus, over-expression of Met protein is dependent on enhanced transcription of a normal *MET* gene, which is a common event in most cases of PTC.

Different intracellular and extracellular mechanisms may lead to increased *MET* transcription. They include activating point mutations in genes involved in the signaling pathway, down-regulation of microRNAs (miRNA) which negatively regulate the expression of *MET*, HGF production resulting in autocrine or paracrine loops, and hypoxia which ultimately causes increased *MET* transcription.

Activation of Met protein triggers a broad spectrum of biological responses including a genetic program known as “invasive growth”. HGF stimulation of PTC cells causes prompt phosphorylation of Met protein, stimulates the migratory/invasive capacity of tumor cells, up-regulates the expression of the pro-invasive molecule cyclo-oxygenase 2, and down-regulates the anti-metastatic protein KAI-1/CD82.

Met protein over-expression in PTC cells profoundly affects the tumor microenvironment by facilitating tumor-associated angiogenesis and by promoting the intratumoral recruitment of inflammatory cells. Taken together all these observations suggest that over-expression of *MET* gene has a central role in the tumorigenesis of papillary carcinoma of the thyroid.
